# Anti-oral Squamous Cell Carcinoma Effects of a Potent TAZ Inhibitor AR-42

**DOI:** 10.7150/jca.32436

**Published:** 2020-01-01

**Authors:** Lingyu Su, Si Wang, Ting Yuan, Xudong Xie, Xiaoming Fu, Ping Ji, Lei Zhong, Wenzhao Liu

**Affiliations:** 1 College of Stomatology, Chongqing Medical University, Chongqing, China;; 2 Personalized Drug Therapy Key Laboratory of Sichuan Province, Sichuan Provincial People's Hospital, School of Medicine, University of Electronic Science and Technology of China, Chengdu, Sichuan, China;; 3 Chongqing Research Center for Oral Diseases and Biomedical Science, Chongqing, China;; 4 Chongqing Municipal Key Laboratory of Oral Biomedical Engineering of Higher Education, Chongqing, China;; 5 Department of Pharmacy, Sichuan Academy of Medical Sciences and Sichuan Provincial People's Hospital, Chengdu, Sichuan, China.

**Keywords:** oral squamous cell carcinoma, AR-42, histone deacetylase, cancer stem cell.

## Abstract

Oral squamous cell carcinoma (OSCC) is one of the most common malignancies worldwide. Although great progress has been made in diagnosis and treatment strategies in recent years, the 5-year survival rate of OSCC patients is still disappointingly low. Hence, there is still an unmet medical need for sufferers with OSCC. As a downstream effector of Hippo pathway, TAZ was up-regulated in multiple cancers including OSCC, and considered as an effective therapeutic target. In this study, we constructed a stable transfected cell line HEK293-TAZ to screen TAZ inhibitor using dual-luciferase reporter assay, and found a potential TAZ inhibitor AR-42. The results showed that AR-42 effectively suppressed the viability and proliferation of OSCC cells, and induced cellular apoptosis and cell cycle arrest in G2/M phase. Moreover, AR-42 potently inhibited cell invasion and the capacity of sphere-forming, as well as the expression of EMT and cancer stem cell related proteins in OSCC cells, exhibiting potential efficacy against OSCC metastasis and self-renewal of oral cancer stem cell. Further mechanism studies showed that AR-42 inhibited the total amount of TAZ and its paralog YAP mainly through blockade of TAZ/YAP transcription and promotion of TAZ/YAP protein degradation. Additionally, the inhibitory effect of AR-42 against TAZ, as well as its anti-OSCC activity could be also observed in SCC9 xenograft model. Taken together, AR-42 deserves to be further studied as a TAZ inhibitor, and is worthy to be further assessed as a potential drug candidate for OSCC treatment.

## Introduction

Oral cancer is one of the most common malignancies worldwide, with 90% of the histological classification being oral squamous cell carcinoma (OSCC) 1, 2. OSCC is associated with high morbidity and mortality. Although great progress has been made in diagnosis and treatment strategies in recent years, the 5-year survival rate of OSCC sufferers, especially in the advanced stage, remains disappointingly low, which is mainly attributed to the poor drug efficacy, metastatic spread and resistance 3, 4. Therefore, it is still significant to find novel therapeutic targets and medicines for clinical treatment of OSCC.

Hippo pathway is initially discovered as an important regulator for organ size control and tissue growth 5, 6. In recent years, mounting evidence has suggested that dysregulation of Hippo signaling leads to proliferation and anti-apoptosis associated with increased cancer risk 6, 7. As a pivotal downstream effector of Hippo signal cascade, transcriptional co-activator with PDZ-binding motif (TAZ) plays a key role in controlling cell proliferation. Elevated expression of TAZ is closely related to the progression and poor clinical outcomes of a variety of human cancers, and TAZ is considered as a promising target for cancer therapy 8-10. TAZ overexpression was also found to be associated with tumor size, pathological grade and cervical lymph node metastasis, as well as unfavorable prognosis of OSCC. TAZ was an oncogene promoting cell proliferation, migration, invasion, epidermal mesenchymal transition (EMT) and chemoresistance in OSCC. Enriched TAZ could also sustain self-renewal potentiality of oral cancer stem cells 11. Meanwhile, the study by Samantha E. Hiemer and colleagues showed that regions of pre-malignant oral tissues exhibited aberrant nuclear TAZ accumulation. TAZ activity promoted proliferation, survival and migration of OSCC cells* in vitro*, and was required for OSCC tumor growth and metastasis *in vivo*. Knockdown of TAZ led to change in the control of gene expression implicated in pro-tumorigenic signaling 12. Therefore, TAZ is also a biomarker and viable druggable target for OSCC therapy.

In this study, we constructed a stable transfected cell line HEK293-TAZ to screen TAZ inhibitor using dual-luciferase reporter assay, and found AR-42, a HDAC inhibitor, had the inhibitory activity against TAZ. This account reports the anti-tumor effects of AR-42 in the treatment of OSCC.

## Materials and Methods

### Dual-luciferase reporter assay

HEK293-TAZ cell line stably incorporating TAZ-dependent firefly luciferase and thymidine kinase promoter-driven renilla luciferase reporters was constructed by co-transfection with lentivirus vector (CV060). The dual-luciferase reporter assay was performed on HEK293-TAZ cells to screen TAZ inhibitor. In brief, HEK293-TAZ cells were seeded in 96-well plates, and treated with small molecules from ChemDiv compound library and an in-house focused library for 24 h at 37 ℃. The activities of cellular firefly and renilla luciferase were then measured using Dual-Luciferase^®^ Reporter Assay System (Promega, USA) according to the manufacturers' protocols. All reporter assays were normalized with renilla luciferase activity.

### Real-time quantitative PCR analysis

Total RNA was extracted from AR-42 treated cells using RNAsimple total RNA kit (TIANGEN, China), and reverse transcribed to cDNA using iScript cDNA Synthesis Kit (Bio-Rad, USA) according to the manufacturer's instructions. Real time PCR was conducted using iQ SYBR Green Supermix (Bio-Rad, USA). Data were normalized with GAPDH. The list of primers and their sequences is given in supplementary Table 1.

### Cell culture and reagents

Human oral squamous cell carcinoma cell lines were obtained from American Type Culture Collection (ATCC) and National Platform of Experimental Cell Resources for Sci-Tech (China). The cells were cultured in DMEM/F12 medium (Gibco, USA) supplemented with 10% FBS (Hyclone, USA), and maintained at 37 ℃ in a humidified atmosphere with 5% CO_2_ according to standard procedures. AR-42 was purchased from Target Molecule Corporation (USA).

### Cell viability assay

MTT assay was used to examine the viability of OSCC cells treated with AR-42 or the positive control cis-diamminedichloroplatinum (II) (DDP). Briefly, OSCC cells were seeded in 96-well plates and cultured overnight at 37 ℃, and then incubated in the absence or presence of agents for 72 h. MTT (5 mg/mL) was added for the last 2 h of incubation, and the absorbances at 570 nm were measured after dissolution with acidified SDS (20%). The IC_50_ values were calculated using GraphPad Prism v5.0 software.

### EdU cell proliferation assay

SCC9 cells were seeded in 96-well plates and cultured overnight at 37 ℃, and then treated with AR-42 or DMSO vehicle for 24 h. The proliferation of OSCC cells was detected by EdU incorporation assay following the manufacturer's instruction (RIBOBIO, China). The images were captured using ArrayScan VTI HCS reader (Thermo Scientific, USA).

### Flow cytometry for apoptosis and cell cycle analysis

For apoptosis analysis, SCC9 cells were seeded in 6-well plates and treated with AR-42 or DMSO vehicle the following day for 36 h. Cells were then stained with Annexin V-FITC and propidium iodide (PI) according to the manufacturer's protocol (KeyGEN Biotech, China). For cell cycle analysis, SCC9 cells growing in 6-well plates were treated with AR-42 or DMSO vehicle for 24 h, and then fixed with 70% ethanol for at least 12 h. Cells were then incubated with propidium iodide (50 μg/mL), RNase (100 μg/mL) and 0.1% Triton X-100 in the dark for 30 min, followed by detection by flow cytometry (Becton Dicknson, USA).

### Transwell invasion assay

The transwell chambers (Millipore, USA) were inserted in 24-well plates and pre-coated with 50 μL diluted BD Matrigel (BD Biosciences, USA). SCC9 cells were suspended in serum-free medium and seeded in the upper chambers, followed by treatment with AR-42 or DMSO vehicle. The outer chambers were filled with the complete growth medium containing 10% fetal bovine serum. After incubating for 24 h at 37 ℃, the non-invaded cells were removed by swabbing the Matrigel in transwell chambers, and the invaded cells were fixed with methanol and stained with crystal violet (0.05%).

### Aldefluor assay

The ALDH-positive OSCC cells in the absence or presence of AR-42 were detected using Aldefluor kit (StemCell Technologies, Canada). Briefly, SCC9 cells were incubated in Aldefluor assay buffer containing ALDH substrate BODIPY-amino acetaldehyde (BAAA) for 45 min at 37 ℃. BAAA was catalyzed to its fluorescent product BODIPY-aminoacetate (BAA); this could reflect the activity of ALDH. ALDH-specific inhibitor diethylaminobenzaldehyde (DEAB) was added in a set of cells during the incubation to serve as negative controls. Propidium iodide was used to identify dead cells. OSCC cells were sorted by a flow cytometer (Becton Dicknson, USA), and the data were analyzed by FlowJo v10.0 software.

### Tumorsphere formation assay

SCC9 cells were washed with serum-free DMEM/F12 medium after trypsinization. Then cells were resuspended in DMEM/F12 medium supplemented with 1:50 B27 (Invitrogen, USA), 20 ng/mL EGF (Peprotech, USA), 10 ng/mL bFGF (Peprotech, USA) and 20 μg/mL insulin (Gibco, USA), and seeded in low adherent 6-well plates at a concentration of 5,000 cells per well. Medium was added every 3 days. When the tumor spheres were formed, they were filtered and digested with StemPro accutase (Gibco, USA), and passaged to evaluate the ability of secondary sphere formation in the absence or presence of AR-42.

### Western blotting

TAZ expression in OSCC cell lines (SCC9, HSC3, SCC15) was detected by protein immunoblot. Cells were lysed in RIPA buffer (Beyotime, China) in the presence of protease inhibitor and phosphatase inhibitor. Equivalent amounts of cell extracts were separated using SDS-PAGE, and transferred to polyvinylidene fluoride membrane, followed by immunoblotting hybridization using TAZ antibody (Abcam, Cambridge, MA, USA). Bands were visualized using the enhanced chemiluminescene system (Millipore, USA). For the SCC9 western blot assay, cells were treated with AR-42 for 24 h, and then lysed with RIPA buffer. The proteins of interest were detected on whole-cell extracts as described above. The primary antibodies were all purchased from Abcam and Cell Signaling Technology, and used at a 1:1,000 dilution. The horseradish peroxidase-conjugated secondary antibodies (Zhong Shan Golden Bridge Bio-technology, China) were used at 1:5,000.

### SCC9 xenograft model

All animal studies were performed in accordance with protocols approved by the Animal Care and Use Committee of Sichuan Academy of Medical Sciences and Sichuan Provincial People's Hospital (Chengdu, Sichuan, China). Each female nude mouse (6-8 weeks) was inoculated subcutaneously with 5×10^6^ SCC9 cells in 100 μL serum-free DMEM/F12 medium. When the mean tumor volume reached 150 mm^3^, mice were randomized into three groups (n = 5). AR-42 was administered at 25 and 50 mg/kg by oral gavage every other day. Tumor volumes (length × width^2^ × 0.52) and the body weight of mice were monitored every 3 days. The inhibition rate of tumor growth was calculated using the following formula: 100 × {1 - [(tumor volume_final_ - tumor volume_initial_) for the compound-treated group] / [(tumor volume_final_ - tumor volume_initial_) for the vehicle-treated group]}.

### Tissue microarray and immunohistochemical staining

A tissue microarray (Wuhan Servicebio, XT16-020), which has 69 OSCC tissues and 10 adjacent non-tumor tissues (ANTT) was stained with TAZ antibody (Abcam, Cambridge, MA, USA). The stained tissue chip was read on Pannoramic MIDI scanner (3D HISTECH, Hungary), and analyzed using Quant center software. Moreover, for the immunohistochemical staining of xenografts, tumor tissues were dissected from the sacrificed BALB/c nude mice after treatment with AR-42 or vehicle, and then fixed with 4% paraformaldehyde and embedded in Paraffin. Tumor sections were subjected to immunostaining with TAZ, Ki67 and TUNEL according to the standard protocols. The representative images were taken under a light microscope (Leica, Germany).

### Statistical analysis

Experiments were performed at least in triplicate and data were presented as mean ± SD. For statistical analysis, Student's t-tests were performed using GraphPad Prism v5.0 software. A p value less than 0.05 was considered statistically significant.

## Results

### TAZ Expression in OSCC and adjacent non-tumor tissues

**Figure 1:**
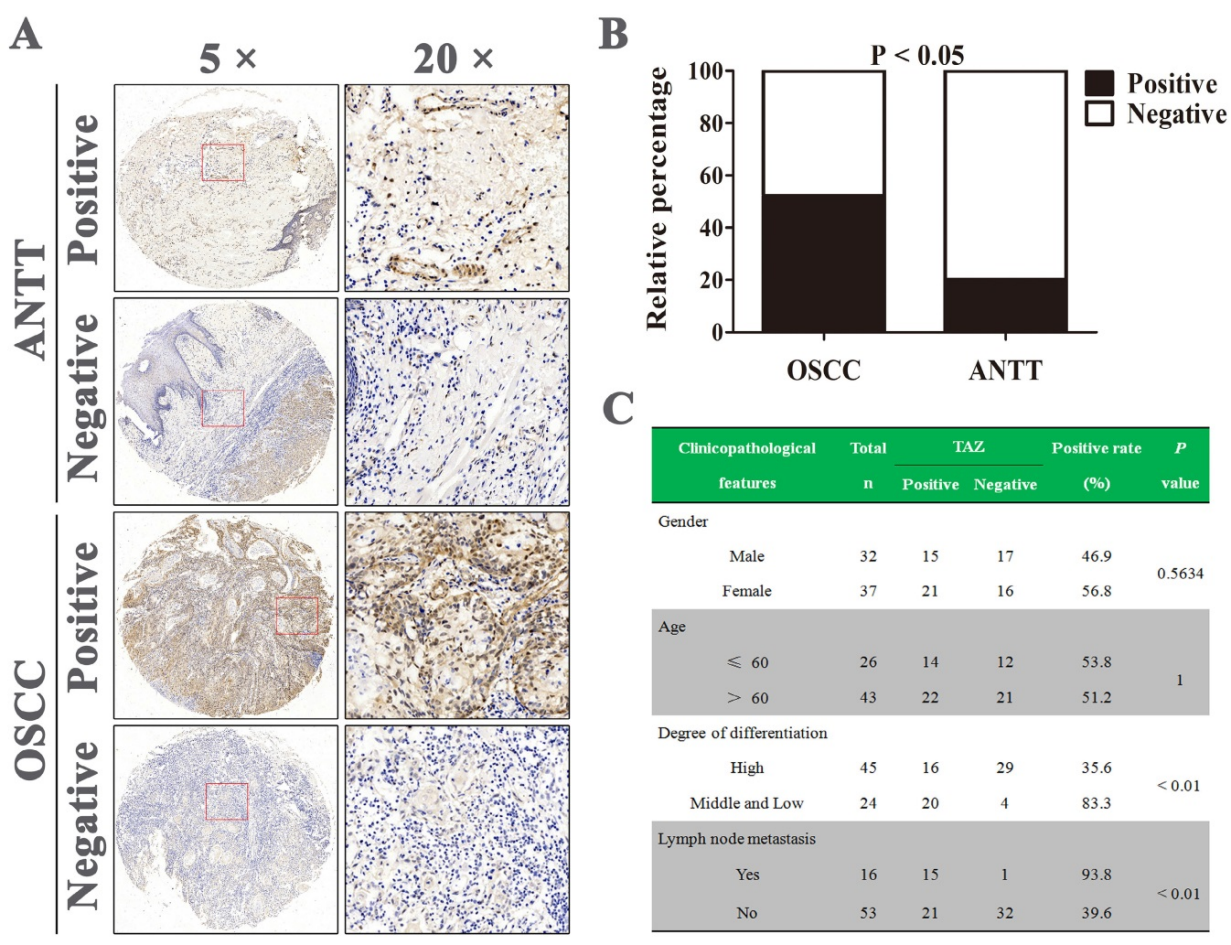
** TAZ expression in human OSCC and adjacent non-tumor tissues**. (A) Representative immunohistochemical staining of TAZ on OSCC and adjacent non-tumor tissues (ANTT). (B) Statistical analysis of positive TAZ expression in human OSCC and ANTT specimens. (C) The relevance of TAZ positive expression with clinicopathological characteristics of OSCC patients.

A tissue microarray containing 69 OSCC tissue specimens and 10 adjacent non-tumor tissues (ANTT) were analyzed by immunohistochemistry. The representative images of TAZ staining in both tissues were shown in Fig. 1A. Positive TAZ expression was identified in approximately 52.2% (36/69) in OSCC tissues and 20% (2/10) in ANTT samples, and there was statistically difference in TAZ expression between OSCC and ANTT (Fig. 1B). Next, we assessed the potential association between TAZ expression and clinicopathological parameters of OSCC patients. As indicated in Fig. 1C, there were no significant correlation between TAZ expression and the gender and age of OSCC sufferers. Noticeably, TAZ positive expression was closely related to medium and low differentiation as well as lymph node metastasis (P < 0.01). These results suggested TAZ might serve as a target for OSCC therapy.

### AR-42 is a potential TAZ inhibitor

**Figure 2:**
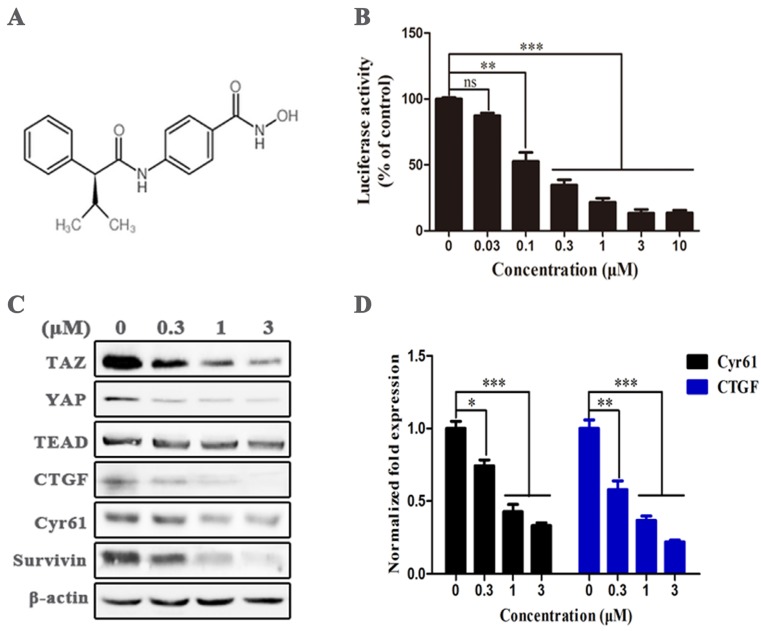
** AR-42 has the ability to inhibit TAZ activity**. (A) The structure of AR-42. (B) The inhibitory activity of AR-42 on HEK293-TAZ cells in the dual-luciferase reporter assay. (C) Western blot analysis of TAZ/YAP and their downstream targets in SCC9 cells after treatment with AR-42. (D) Expression of *CYR61* and *CTGF* at gene level in AR-42 treated SCC9 cells. Column, mean; bars, SD (n=6); *, *P* < 0.05 *vs.* vehicle; **, *P* < 0.01 *vs.* vehicle; ***, *P* < 0.001 *vs. v*ehicle; ns, no significant difference.

We performed dual-luciferase reporter assay on HEK293-TAZ cells to screen TAZ inhibitor in an in-house focused library and ChemDiv compound library, and found that the HDAC inhibitor AR-42 (Fig. 2A) significantly restrained the normalized activity of TAZ promotor-dependent firefly luciferase in a dose-dependent manner (Fig. 2B), suggesting AR-42 was a potential TAZ inhibitor. Immunoblot was further carried out to assess the inhibitory effect of AR-42 at protein level. As indicated in Fig. 2C, AR-42 effectively reduced the expression of TAZ and its downstream targets CTGF, Cyr61, and Survivin at concentrations higher than 0.3 μM. AR-42 could also down-regulate the expression of Yes-associated protein (YAP), another downstream effector of the Hippo pathway, but had no inhibitory activity against the transcription factor TEAD (Fig. 2C). Moreover, the down-regulation of AR-42 against the downstream genes including *CYR61* and *CTGF* was also observed at gene level (Fig. 2D). Taken together, these results indicated that AR-42 was a potent TAZ inhibitor.

### Anti-OSCC effects of AR-42 *in vitro*

**Figure 3:**
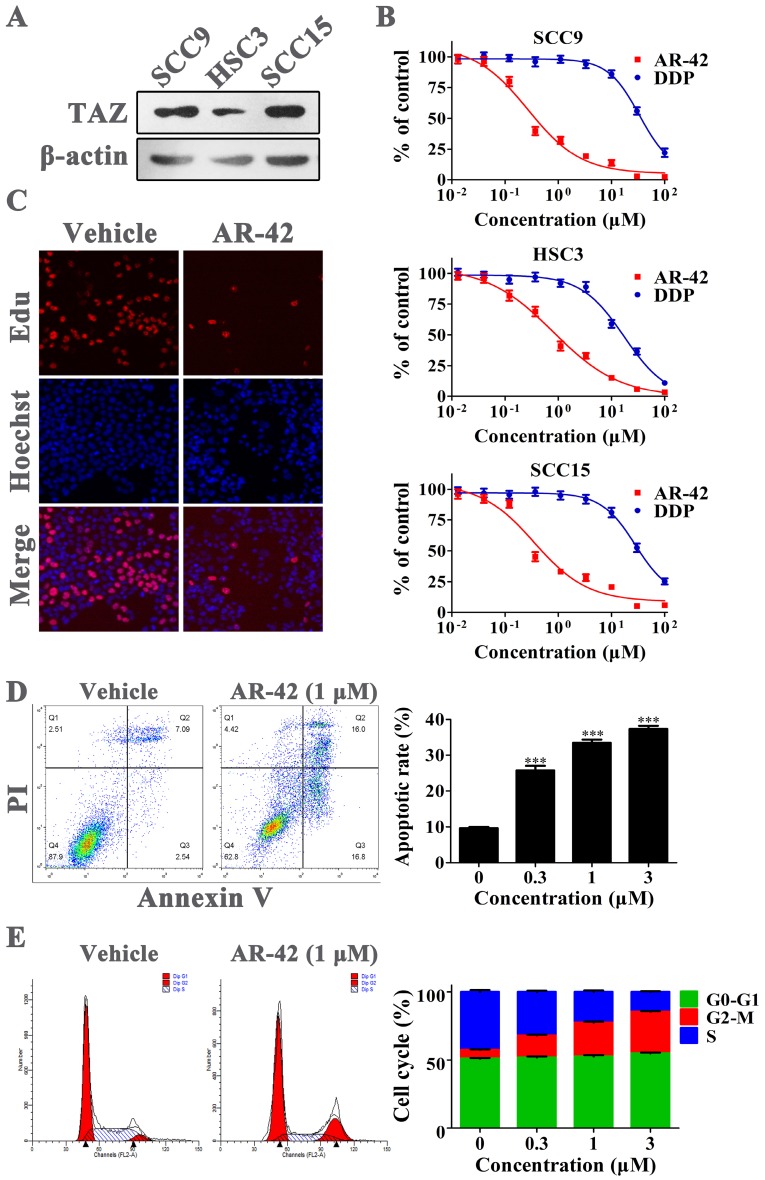
** Anti-OSCC effects of AR-42 *in vitro***. (A) TAZ expression in OSCC cell lines. (B) Anti-viability assay of AR-42 against OSCC cell lines. Points, mean values; bars, SD. (C) Edu incorporation assay on SCC9 cells after treatment with 1 μM AR-42 for 24 h. (D) Annexin V-FITC/PI apoptosis detection on SCC9 cells after treatment with serial dilutions of AR-42 for 36 h. The assays are performed in triplicate, and the percentage of Annexin V-positive cells is quantified for apoptotic rate statistics. ***, *P* < 0.001 *vs* vehicle. (E) Cell cycle profiles of AR-42 treated SCC9 cells. The statistical analysis of cell cycle is presented as means ± SD from three independent experiments.

We initially assessed the protein abundance of TAZ in three OSCC cell lines by western blot. The results showed that TAZ expression in SCC9 and SCC15 cell lines was much higher compared with that in HSC3 cells (Fig. 3A). The former two cell lines were also more sensitive to AR-42 in MTT assay; the IC_50_ values of AR-42 for SCC9, HSC3 and SCC15 cells were 0.24 μM, 0.80 μM and 0.35 μM, respectively (Fig. 3B). As a clinical chemotherapy drug for OSCC, cisplatin (DDP) had lower inhibitory activity on OSCC cells than AR-42, with IC_50_ values of 33.58 μM, 17.30 μM and 28.17 μM for SCC9, HSC3 and SCC15, respectively (Fig. 3B). To investigate the specific anti-OSCC effects of AR-42, cellular proliferation, apoptosis and cell cycle assays were further carried out on SCC9 cells. As shown in Fig. 3C, AR-42 at the concentration of 1 μM significantly reduced the number of proliferating cells (red nuclei) compared with DMSO vehicle group. In addition, the results of flow cytometry demonstrated that AR-42 could dose-dependently increase the Annexin V-positive populations, with apoptotic rate of 25.7%, 33.5% and 37.4% for 0.3 μM, 1 μM, and 3 μM treatment groups, respectively (Fig. 3D). Meanwhile, the cell-cycle arrest in G2/M phase was also observed in AR-42 treated cells (Fig. 3E). These data suggested that AR-42 exerted anti-OSCC effects through inhibition of proliferation, and induction of apoptosis and cell-cycle arrest.

### AR-42 inhibits OSCC cell invasion and EMT phenotype

**Figure 4:**
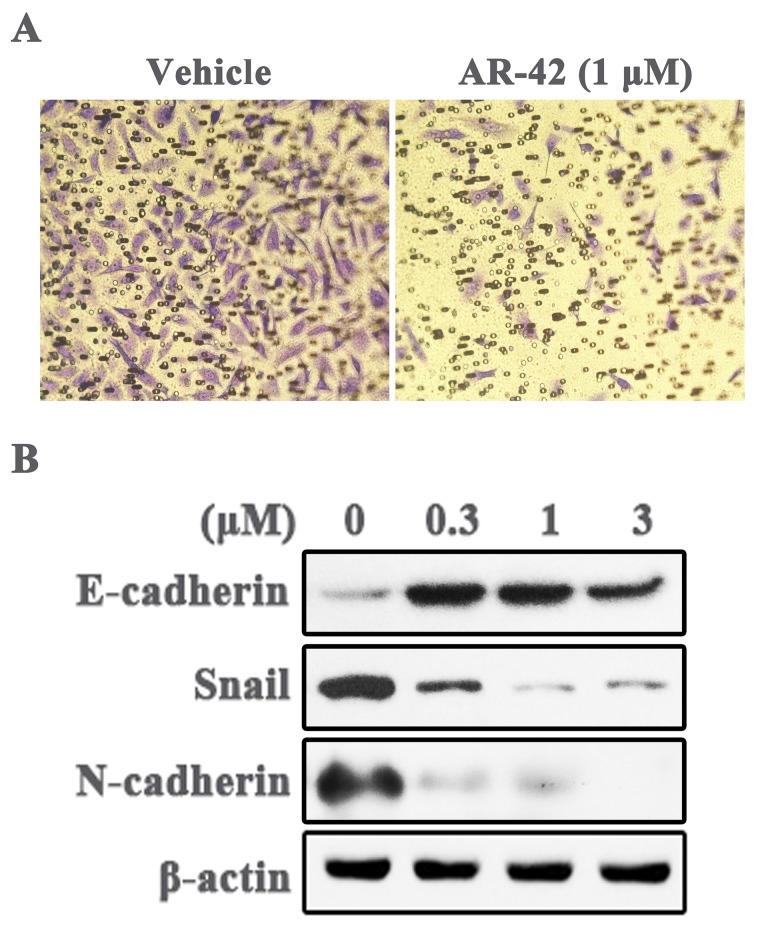
** AR-42 inhibits invasion and EMT phenotype of SCC9 cells**. (A) The representative images (40×) of SCC9 transwell invasion assay in the absence or presence of AR-42 (1 μM). (B) Western blot analysis of the expression of EMT-associated proteins in SCC9 cells treated with AR-42.

Metastasis is the leading cause of cancer progression, and TAZ up-regulation is closely related to tumor metastasis. Therefore, we assessed the ability of AR-42 in inhibiting cell invasion, a pivotal step of tumor metastasis, by transwell invasion assay. As depicted in Fig. 4A, the number of invading SCC9 cells was markedly diminished by 1 μM AR-42 as compared with vehicle. Moreover, epithelial mesenchymal transition (EMT) is a necessary stage in the process of tumor metastasis. We further detected the expression of several EMT related proteins in AR-42 treated SCC9 cells. The results showed that AR-42 up-regulated the epithelial marker E-cadherin, and decreased the expression of mesenchymal marker N-cadherin, as well as the EMT-related transcription factor Snail (Fig. 4B). To sum up, these data demonstrated that AR-42 also had potential activity to inhibit OSCC metastasis.

### AR-42 exhibits anti-cancer stem cell activity in OSCC cells

**Figure 5:**
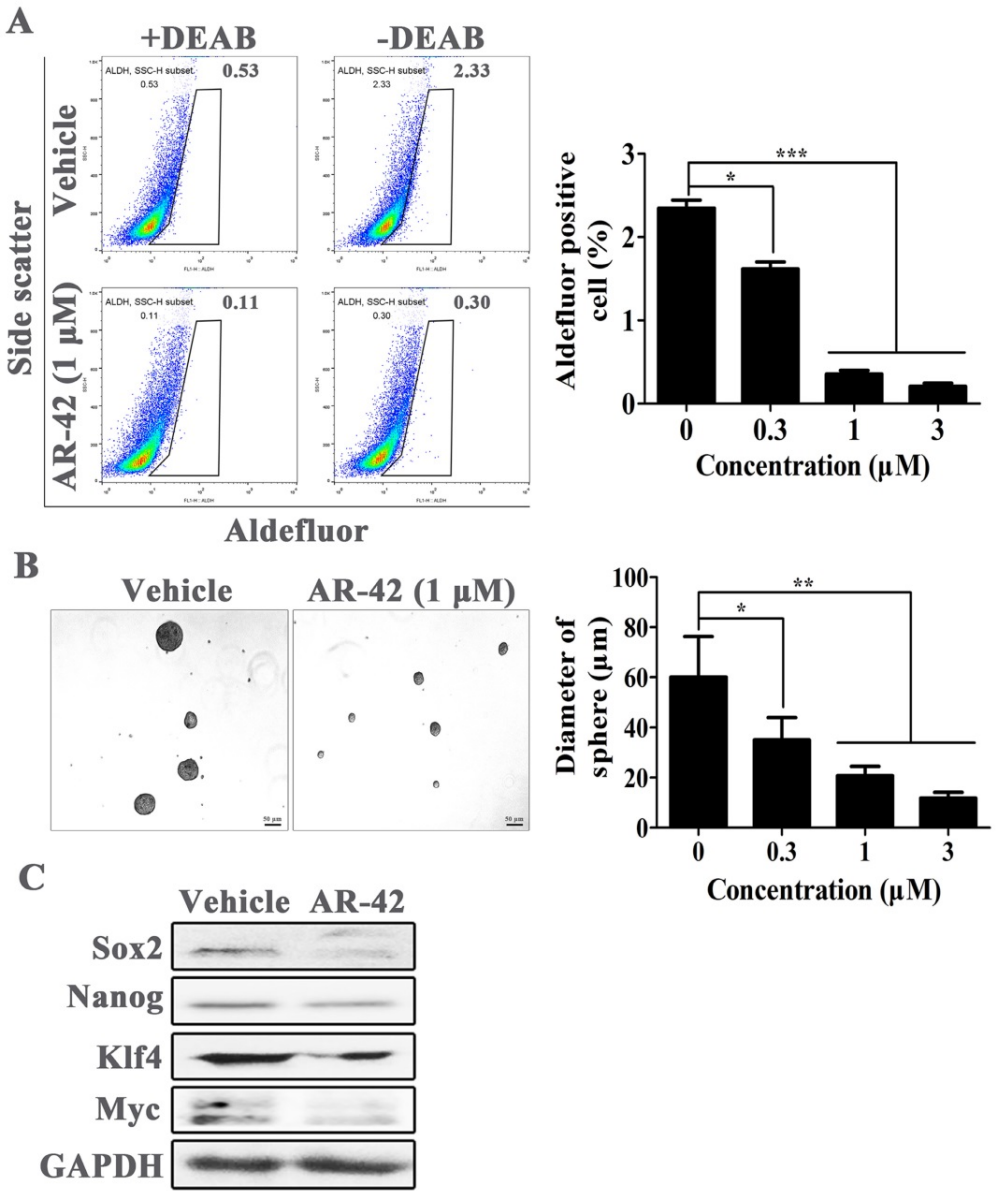
** AR-42 inhibits the phenotype of OSCC stem cells**. (A) ALDH^+^ cells detected in SCC9 cells by flow cytometry analysis. Baseline fluorescence was established by inhibiting ALDH activity with DEAB (left) and used to generate a gate that will identify ALDH^+^ populations in SCC9 cells incubated without DEAB (right). The ALDH^+^ cells incubated without DEAB were used for statistical analysis. (B) Representative images of secondary sphere-forming assay of SCC9 spheroids. Sphere diameters in different treatment group were used for statistical analysis. (C) Expression of cancer stem cell-associated proteins in AR-42 treated SCC9 cells. Column, mean; bars, SD (n=6); *, *P* < 0.05 *vs.* vehicle; **, *P* < 0.01 *vs.* vehicle; ***, *P* < 0.001 *vs.* vehicle.

TAZ was considered as a pivotal protein for the maintenance of cancer stem cell. Hence, the anti-cancer stem cell activity of AR-42 was further evaluated in OSCC cells. We used Aldefluor assay followed by FACS analysis to assess the amount of cell populations with ALDH1 enzymatic activity; ALDH1 is a specific cancer stem cell marker for various tumors including OSCC. As shown in Fig. 5A, SCC9 cell line had an average of 2.3% ALDH1-positive cells. However, the ALDH1-positive populations were significantly reduced after treatment with AR-42, with positive rates of 1.6%, 0.35% and 0.21% for 0.3 μM, 1 μM, and 3 μM treatment groups, respectively. Furthermore, we assessed the secondary sphere-forming capacity of SCC9 cells in the absence or presence of AR-42, and found AR-42 treated spheres were all smaller in size than that in vehicle group (Fig. 5B). Meanwhile, the expression of cancer stem cell associated proteins, such as Sox2, Nanog, Klf4 and Myc, were also abated at different levels by AR-42 at 1 μM (Fig. 5C). Taken together, these results indicated that AR-42 could inhibit self-renewal of OSCC stem cell.

### Mechanisms of AR-42 against TAZ expression

**Figure 6:**
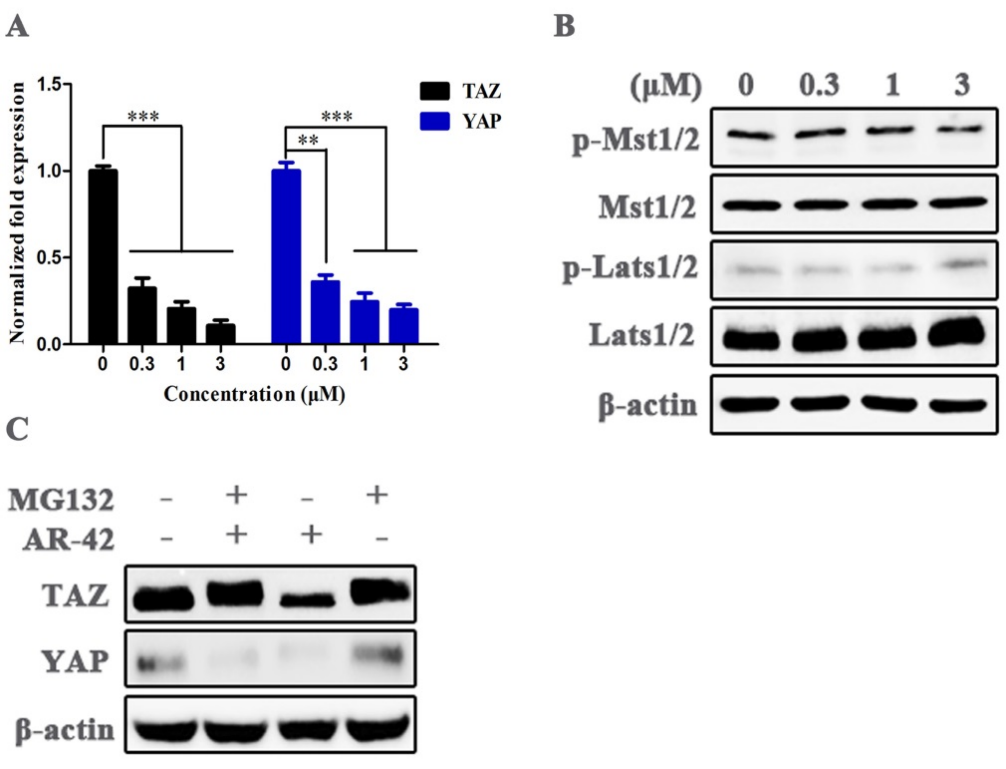
** Mechanisms of action of AR-42 against TAZ.** (A) mRNA expression of *TAZ* and *YAP* in AR-42 treated SCC9 cells. Column, mean; bars, SD (n=6); **, *P* < 0.01 *vs.* vehicle; ***, *P* < 0.001 *vs. v*ehicle. (B) Influence of the central kinase cascades of Hippo pathway after treatment with AR-42 in SCC9 cells. (C) AR-42 induced the proteasome-dependent degradation of TAZ. SCC9 cells were treated with AR-42 for 24 h in the presence or absence of 10 μM proteasome inhibitor MG132.

We analyzed the anti-TAZ mechanisms of AR-42 in transcription and protein levels. The results of qRT-PCR showed that mRNA levels of TAZ, as well as its paralog YAP, were significantly reduced in AR-42 treated cells compared with vehicle group (Fig. 6A). We further detected the activities of central kinase cascades of Hippo pathway in SCC9 cells after treatment with AR-42, and no distinct changes were observed in the phosphorylation of Mst1/2 and Lats1/2, as well as their total amounts in the absence or presence of AR-42 (Fig. 6B), suggesting the inhibition of TAZ/YAP was not attributed to the increased activity of the upstream kinases of Hippo signaling. Moreover, as indicated in Fig. 6C, treatment with 1 μM AR-42 evidently decreased the total amount of TAZ and YAP in SCC9 cells, while the reduction of TAZ (but not YAP) was canceled by a proteasome inhibitor MG-132, indicating that AR-42 could also facilitate the degradation of TAZ by the ubiquitin-proteasome system. These results displayed that AR-42 inhibited TAZ/YAP expression mainly through blockade of TAZ/YAP transcription and promotion of TAZ/YAP protein degradation.

### Antitumor efficacy of AR-42 in SCC9 xenograft model

**Figure 7:**
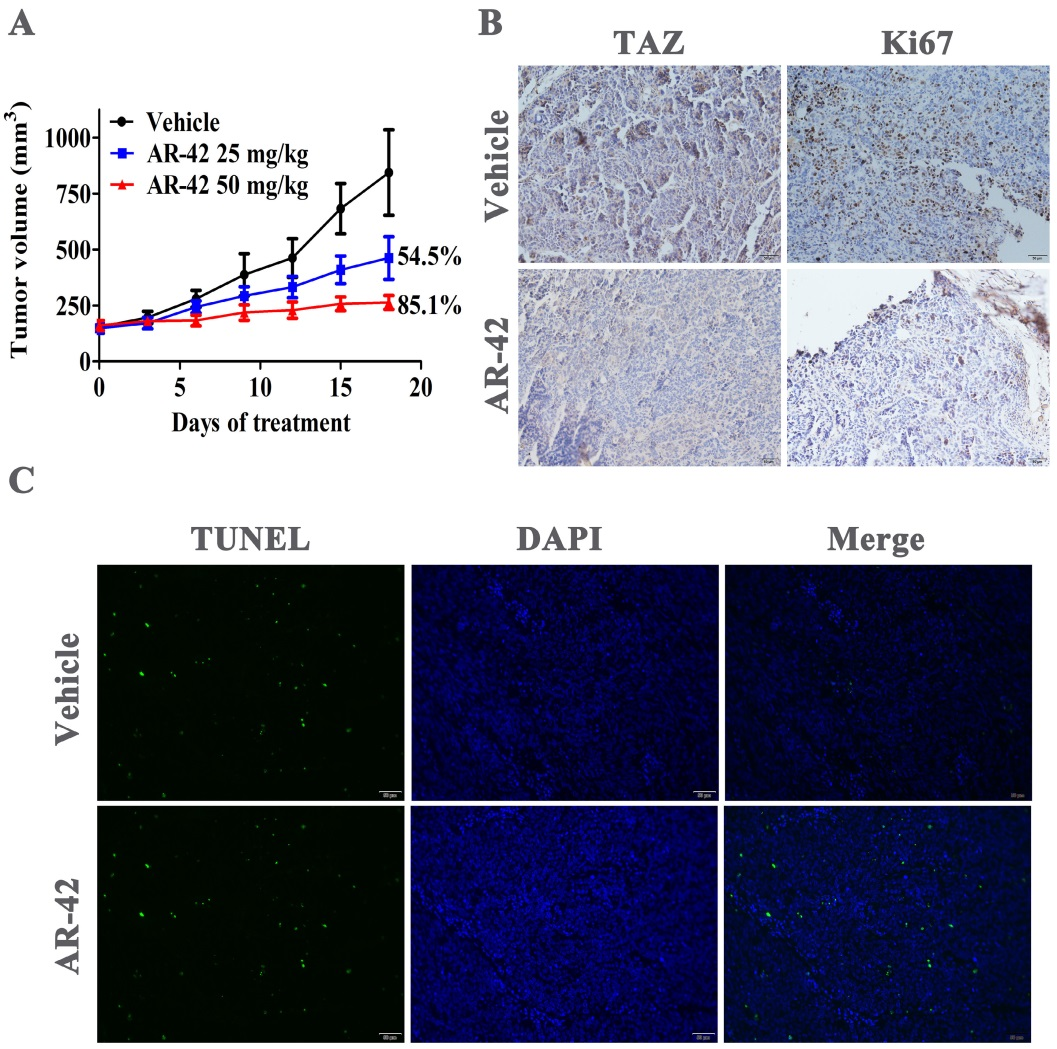
** Anti-OSCC activity of AR-42* in vivo***. (A) Tumor volumes of SCC9 xenograft model. Points, mean tumor volume; bars, SD (n=5). (B) TAZ and Ki67 immunohistochemical staining of SCC9 xenograft tumors from vehicle group and AR-42 at 50 mg/kg. Scale bar represents 50 μm. (C) TUNEL staining was used to detect the apoptosis of tumor tissues from vehicle control and 50 mg/kg AR-42 treatment groups in SCC9 model. Scale bar represents 50 μm.

We further assessed the* in vivo* anti-tumor effect of AR-42 in SCC9 xenograft model. Oral administrations of AR-42 at 25 and 50 mg/kg potently inhibited tumor growth in a dose-dependent manner with tumor growth inhibition rates of 54.5% and 85.1%, respectively (Fig. 7A). No significant weight loss was observed in AR-42 treated groups compared with vehicle group (data not shown). Additionally, as shown in Fig. 7B, AR-42 at 50 mg/kg evidently restrained TAZ expression *in vivo*. Meanwhile, the immunohistochemical assays showed that AR-42 could also lead to a significant decrease in Ki67-positive tumor cells (proliferating cells) and substantial increase in TUNEL-positive tumor cells (apoptotic cells) in xenograft model (Fig. 7B and 7C). To sum up, AR-42 could also inhibit the expression of TAZ *in vivo*, thus exerting its effects of anti-proliferation and pro-apoptosis in SCC9 xenograft.

## Discussion

The Hippo pathway features a central kinase cascade formed by the serine/threonine kinases Mst1/2 and Lats1/2, whose activations lead to phosphorylation, cytoplasmic retention and degradation of the downstream transcriptional co-activators TAZ and its paralog YAP, and eventually prevent the transactivation of the DNA binding transcriptional factor TEADs in mammals 13-16. Deregulation of Hippo cascade leads to the nucleus accumulation of TAZ/YAP, which binds to and activates the transcription factors TEADs, and activates multiple cellular functions related to cell proliferation, apoptosis, migration and differentiation 5, 17, 18. Hence, Hippo cascade is a tumor suppressor pathway, and TAZ/YAP acts as an oncogene in various cancers including OSCC 12, 19-22. The results of this study exhibited that positive TAZ expression in OSCC tissues was greatly higher compared to that in ANTT specimens, and the elevated TAZ expression was closely related to medium and low differentiation as well as lymph node metastasis of OSCC, further supporting TAZ as a potential therapeutic target for OSCC. Moreover, we screened a small molecular TAZ inhibitor AR-42, and evaluated its anti-tumor effects in OSCC. AR-42 effectively suppressed the viability and proliferation of OSCC cells, and induced cellular apoptosis and cell cycle arrest in G2/M phase. More importantly, AR-42 also potently inhibited cell invasion, the capacity of sphere-forming, as well as the expression of EMT and cancer stem cell related proteins in OSCC cells, exhibiting potential efficacy against OSCC cell metastasis and self-renewal of oral cancer stem cells. Meanwhile, the inhibitory effect of AR-42 against TAZ, as well as its anti-OSCC activity could also be observed in SCC9 xenograft model.

As the paralog of TAZ, YAP also acts as a downstream effector of Hippo pathway, and is regulated in a manner similar to TAZ. Elevated YAP expression was also observed in OSCC tissues compared with adjacent normal tissues, and YAP was a crucial regulator that exerted pro-proliferation and anti-apoptosis effects in OSCC through actions affecting cell cycle and intrinsic apoptotic signaling 23. Furthermore, YAP was found conferring resistance to cisplatin in human OSCC cells 22. On account of the vital function of YAP in the progression of OSCC, the anti-YAP activity of AR-42 was also assessed. AR-42 effectively inhibited the total amount of YAP in both transcription and protein levels, while the mechanism was slightly different from that of TAZ; AR-42 induced a proteasome-dependent degradation of TAZ protein (Fig. 6C).

AR-42 screened from the positive compound library is a HDAC inhibitor. HDACs regulate the acetylation status of histones and multiple non-histone substrates, and they are themselves therapeutic targets for cancer 24-26. Recently, there are many HDAC inhibitors undergoing clinical trials, and even approved for cancer therapy in clinic 26-28. Thus, it is difficult to exactly differentiate how much of the observed efficacy of AR-42 are due to the inhibition of HDACs and how much due to its activity against TAZ/YAP. Roughly speaking, TAZ/YAP suppression should play more important roles on the anti-OSCC effects, especially in inhibiting the EMT and cancer stem cell-like phenotypes of OSCC cells; this speculation was based on the vital function of TAZ/YAP in OSCC progression and the high activity of AR-42 against TAZ/YAP. It is worth mentioning that not all HDAC inhibitors show anti-TAZ activity in the screening performed on HEK293-TAZ cells (data not shown), indicating there is little correlation between the anti-TAZ/YAP effects of AR-42 and its potency against HDACs.

In summary, the present study screened a high active TAZ inhibitor AR-42 through dual-luciferase reporter assay performed on HEK293-TAZ cells. Further pharmacodynamic studies showed that AR-42 could not only significantly inhibit the growth of OSCC cells, but also had the ability to depress the phenotypes of EMT and oral cancer stem cell. AR-42 deserves to be further studied as a TAZ inhibitor, and is also worthy to be further assessed as a potential drug candidate for OSCC treatment.

## Supplementary Material

Supplementary tables.

## Abbreviations

OSCC: oral squamous cell carcinoma
HDAC: histone deacetylase
TAZ: transcriptional co-activator with PDZ-binding motif
YAP: yes-associated protein
EMT: epithelial mesenchymal transition
