# Supplementary material for: Anti-oral Squamous Cell Carcinoma Effects of a Potent TAZ Inhibitor AR-42
Source: J Cancer. 2020 Jan 1;11(2):364–73. doi: 10.7150/jca.32436 (PMC6930442; doi:10.7150/jca.32436)
Supplement: Supplementary file 1 — Supplementary tables. [file jcav11p0364s1.pdf]

Anti-oral Squamous Cell Carcinoma Effects of a Potent TAZ Inhibitor AR-42

Lingyu Su[1,3,4], Si Wang[1,3,4], Ting Yuan[2,5], Xudong Xie[1,3,4], Xiaoming Fu[1,3,4], Ping Ji[1,3,4], Lei Zhong[2,5], Wenzhao Liu[1,3,4]

1. College of Stomatology, Chongqing Medical University, Chongqing, China

2. Personalized Drug Therapy Key Laboratory of Sichuan Province, Sichuan Provincial People's Hospital, School of Medicine, University of Electronic Science and Technology of China, Chengdu, Sichuan, China

3. Chongqing Research Center for Oral Diseases and Biomedical Science, Chongqing, China

4. Chongqing Municipal Key Laboratory of Oral Biomedical Engineering of Higher Education, Chongqing, China

5. Department of Pharmacy, Sichuan Academy of Medical Sciences and Sichuan Provincial People's Hospital, Chengdu, Sichuan, China

Corresponding authors: Dr. Wenzhao Liu, Chongqing Medical University, No. 426 North Songshi Road, Chongqing 401147, China. Email: liuwenzhao@126.com. Dr. Lei Zhong, Department of Pharmacy, Sichuan Academy of Medical Sciences and Sichuan Provincial People's Hospital, No. 32 West Second Section First Ring Road, Chengdu 610072, Sichuan, China. Email: zhonglei@med.uestc.edu.cn. Dr. Ping Ji, College of Stomatology, Chongqing Medical University, No. 426 North Songshi Road, Chongqing 401147, China. Email: Jiping_62@hotmail.com.

Table S1 Primers used for qRT-PCR

Gene	Forward primer	Reverse primer
TAZ	GGCTGGGAGATGACCTTCAC	CTGATGGGGTGGTTCTCT
YAP	ACCCTCGTTTTGCCATGAAC	TGTGCTGGGATTGATATTCCGTA
CTGF	AGGAGTGGGTGTGTGACGA	CCAGGCAGTTGGCTCTAATC
Cyr61	AGCCTCGCATCCTATACAACC	TTCTTTCACAAGGCGGCACTC
GAPDH	TGGAAGGACTCATGACCACA	TTCAGCTCAGGGATGACCTT
